# Genetic model of UBA5 deficiency highlights the involvement of both peripheral and central nervous systems and identifies widespread mitochondrial abnormalities

**DOI:** 10.1093/braincomms/fcad317

**Published:** 2023-11-20

**Authors:** Rita J Serrano, Viola Oorschot, Dashika Palipana, Vanessa Calcinotto, Carmen Sonntag, Georg Ramm, Robert J Bryson-Richardson

**Affiliations:** School of Biological Sciences, Monash University, Melbourne 3800, Australia; Monash Ramaciotti Centre for Cryo-Electron Microscopy, Monash University, Melbourne 3800, Australia; School of Biological Sciences, Monash University, Melbourne 3800, Australia; School of Biological Sciences, Monash University, Melbourne 3800, Australia; School of Biological Sciences, Monash University, Melbourne 3800, Australia; Monash Ramaciotti Centre for Cryo-Electron Microscopy, Monash University, Melbourne 3800, Australia; Department of Biochemistry and Molecular Biology, Biomedicine Discovery Institute, Monash University, Melbourne 3800, Australia; School of Biological Sciences, Monash University, Melbourne 3800, Australia

**Keywords:** UBA5, ufmylation, encephalopathy, mitochondria, zebrafish

## Abstract

Variants in *UBA5* have been reported to cause neurological disease with impaired motor function, developmental delay, intellectual disability and brain pathology as recurrent clinical manifestations. *UBA5* encodes a ubiquitin-activating-like enzyme that activates ufmylation, a post-translational ubiquitin-like modification pathway, which has been implicated in neurodevelopment and neuronal survival. The reason behind the variation in severity and clinical manifestations in affected individuals and the signal transduction pathways regulated by ufmylation that compromise the nervous system remains unknown. Zebrafish have emerged as a powerful model to study neurodegenerative disease due to its amenability for *in vivo* analysis of muscle and neuronal tissues, high-throughput examination of motor function and rapid embryonic development allowing an examination of disease progression. Using clustered regularly interspaced short palindromic repeats-associated protein 9 genome editing, we developed and characterized zebrafish mutant models to investigate disease pathophysiology. *uba5* mutant zebrafish showed a significantly impaired motor function accompanied by delayed growth and reduced lifespan, reproducing key phenotypes observed in affected individuals. Our study demonstrates the suitability of zebrafish to study the pathophysiology of *UBA5*-related disease and as a powerful tool to identify pathways that could reduce disease progression. Furthermore, *uba5* mutants exhibited widespread mitochondrial damage in both the nervous system and the skeletal muscle, suggesting that a perturbation of mitochondrial function may contribute to disease pathology.

## Teaser

Serrano *et al*. describe zebrafish models for investigating UBA5-related disease pathophysiology. *uba5* mutants show locomotor impairment, cerebellar degeneration, delayed growth and reduced survival, reproducing phenotypes observed in affected individuals. Furthermore, involvement of the peripheral nervous system and widespread mitochondrial damage at early stages of the phenotype were identified.

## Graphical Abstract

**Graphical Abstract:**
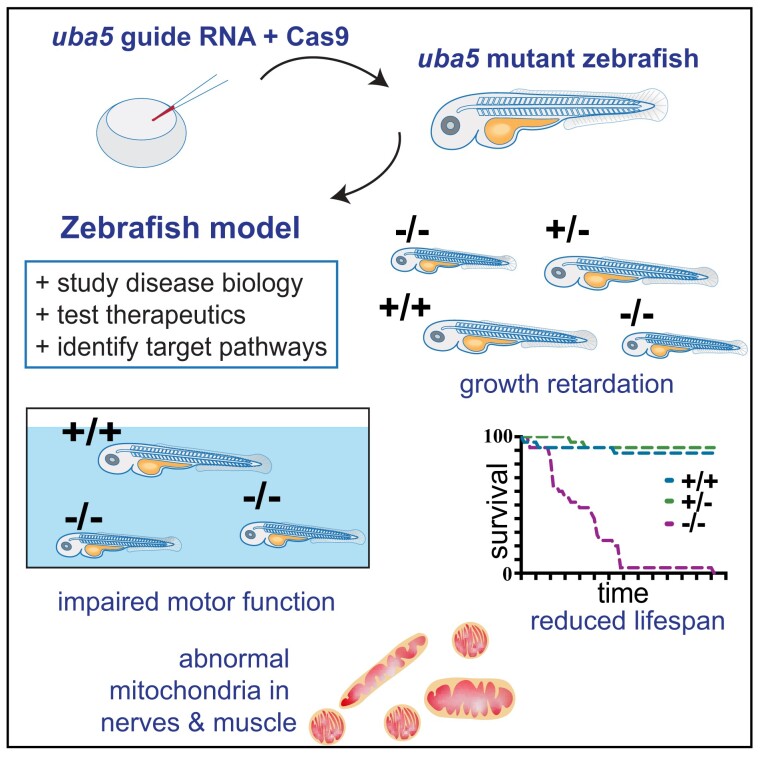

## Introduction

To date, 38 affected individuals with variants in ubiquitin-like activating enzyme 5 (*UBA5*) have been described, and they present with 1 of 3 closely related phenotypes, severe infantile–onset encephalopathy with or without seizures, severe congenital neuropathy, or childhood-onset cerebellar atrophy with ataxia. Affected individuals with severe infantile–onset encephalopathy exhibit a range of clinical symptoms, including mild to severe intellectual disability, progressive microcephaly, progressive growth failure, poor muscle tone, movement disorder, infantile spasms and/or refractory seizures. Brain imaging frequently does not reveal abnormalities at the onset of disease; however, non-specific findings such as mildly delayed myelination, white matter hyperintensities, thinning of the thalamus and the corpus callosum, cerebellar hypoplasia or generalized brain atrophy are detected as the disease progresses.^1-10^ Cerebellar ataxia–affected individuals show a milder phenotype, displaying cerebellar atrophy, gait ataxia, dysarthria and delayed growth during childhood, with mild demyelinating sensory-motor peripheral neuropathy reported in one case.^11^ More recently, Cabrera-Serrano *et al*.^12^ identified five individuals with severe congenital neuropathy associated with reduced fetal movements and severe sensory-motor peripheral neuropathy, with no central nervous system involvement. The differing involvement of the peripheral and central nervous system in the three clinical presentations of the disease is not understood. Individuals with the most severe forms of the disease die soon after birth or during infancy;^4,8,12^ however, individuals with milder forms have been reported to survive past 20 years of age.^9,11^

Affected individuals described to date carry either two missense variants or a loss-of-function variant associated with either a hypomorphic variant or a missense variant, in *UBA5*.^1-12^ The cause of the variance in clinical severity and symptoms observed in affected individuals remains to be demonstrated; however, it is hypothesized to result from differential residual UBA5 activity.^5,8,9,11,12^ Colin *et al*.^5^ noted that seizures resistant to anti-seizure medication are often associated with at least one null variant, resulting in significantly reduced UBA5 activity, while developmental delay and impaired motor function, but not seizures, are observed in individuals with two missense variants that cause milder UBA5 activity loss.

*UBA5* encodes a ubiquitin-like activating enzyme (E1) for ufmylation, a pathway for post-translational modification of proteins.^13^ UBA5 binds and activates UFM1 (ubiquitin-like), which forms a bond with the E2 conjugating enzyme (UFC1) and then complexes with the E3 ligase (UFL1), resulting in the addition of UFM1 to target proteins. In humans, there are two UBA5 isoforms, comprised of an adenylation domain that includes the ATP-binding domain and the cysteine active site, the UFM1-interacting domain and the UFC1-binding domain, with a 56 amino acid extension at the N-terminus in the longer isoform.^14^ The N-terminal extension enhances ATP binding to UBA5, critical for UFM1 activation, and promotes the transfer of UFM1 to UFC1.^15^ While most variants are found in compound heterozygosity and are located within the adenylation or UFC1-binding domain, leading to a partial loss of function or delayed UBA5 activity,^5,9,11^ homozygous variants within the N-terminal domain have been described to lead to a significant reduction in UBA5-UFM1 complexes and cause the most severe forms of disease.^8,12^

Ufmylation has been previously implicated in neuronal development and homeostasis. Ufc1 and Ufm1 are known to interact and co-localize with NCAM1, a neural cell adhesion molecule involved in neuronal cell differentiation.^16^ Muona *et al*.^9^ demonstrated that brain-specific knockout of *Ufm1* in mice results in microcephaly and neuronal apoptosis. Moreover, mutations in *UFM1* and *UFC1* were shown to cause neurologic disease with global developmental delay, progressive microcephaly, short stature and refractory epilepsy,^17,18^ a phenotype highly reminiscent of that observed in individuals with *UBA5*-related epileptic encephalopathy. Despite these findings, the molecular events resulting from *UBA5* deficiency and impaired ufmylation causing the neurological disturbances are yet to be determined, and there are no effective treatments available to affected individuals.

Previous studies with animal models have shown that a complete loss of Uba5 is lethal during *Drosophila* and mouse embryonic development,^11,19^ not allowing the manifestation and study of the pathophysiology associated with disease. A knockdown of *Uba5* in *Drosophila*, however, recapitulated some of the features of UBA5 deficiency, including reduced motor function and shortened lifespan. The locomotive defects were accompanied by a reduced number, but an increased size, of synaptic boutons at the neuromuscular junction, indicating abnormal synaptic formation and transmission. However, the locomotive deficits and neuromuscular junction aberrations were mild,^11^ suggesting a minor disturbance of the nervous system.

Zebrafish have emerged as a valuable model to study neural disorders, exhibiting conserved neuronal networks and signalling pathways to the human brain. Zebrafish have the advantage of being highly amenable to *in vivo* examination of neural development and degeneration due to their external, and transparent, development. Their swimming behaviour is easily examined from early embryo to adult developmental stages using automated tracking systems.^20,21^ Furthermore, the large number of offspring makes the zebrafish model suitable for high-throughput screening of potential therapeutics for neural disorders, as described previously.^22^

Previous work has shown that *uba5* morphant zebrafish exhibit reduced motor function and abnormal movement pattern reminiscent of the seizure-like episodes observed in affected individuals,^5^ demonstrating that zebrafish are suited to investigation of the pathogenesis associated with *UBA5* deficiency. However, morpholino knockdown does not allow an examination of the progression in neuronal damage and motor and growth deficiencies, which occur in affected individuals. To examine disease progression, we generated and examined zebrafish *uba5* mutant models. We show that *uba5^−/−^* animals display impaired motor function, delayed growth and reduced survival, recapitulating phenotypes observed in affected individuals. Together, these results validate these new Uba5 deficiency models for the investigation of the cellular and molecular mechanisms and for testing of potential therapeutics. Using these models, we further demonstrate that *uba5^−/−^* animals exhibit peripheral nerve and cerebellar axonal damage at early stages of the phenotype and identified mitochondrial pathology in both the peripheral and the central nervous systems, and in the skeletal muscle. These findings highlight the contribution of both the peripheral and the central nervous systems to the disease and suggest that mitochondria damage might underlie the neuronal dysfunction.

## Materials and methods

### Zebrafish maintenance, sgRNA injections and genotyping

Animal work was conducted in accordance with the care and use of animals for scientific purposes approved by Monash Animal Ethics Committees (MARP/SOP0082 and MARP/SOP0081). The generation of CRISPR mutant strains was approved under application BSCI201507, and the long-term analysis of locomotion, pathology and survival was carried out under application BSCI201618. Fish were maintained in breeding colonies, approval MARP2015/004/BC.

To create CRISPR mutant strains, target sites within the first and third exons of *uba5* were selected in accordance with what was described in the study by Montague *et al.*^23^ sgRNAs synthesis was completed using the High Scribe T7 High Yield RNA Synthesis Kit (NEB). sgRNAs (150 ng/μl) were co-injected with the Cas9 protein (800 ng/μl; PNA Bio) and cascade blue-labelled dextran (Molecular Probes) into one-cell wild-type TU embryos. At 1 dpf, embryos were sorted for Cascade Blue labelling.

Genotyping was performed with primers flanking the target regions. The primers used for genotyping were ex1F: GAGACACTCATCCTCGCCAGCTG, ex1R: CTACGACTTCGGC GCTCATTTGCT, ex3F: GAGAATGGGGATAGTGCAAGAC and ex3R: TGGTGCTGGG TAATATGACAAA. DNA amplification was performed using a 35-cycle protocol with denaturation at 95°C, annealing at 58°C and extension at 72°C. A single polymerase chain reaction (PCR) reaction (10 μl) consisted of 1 μl DNA (20–50 ng/μl concentration), 5 μl GoTaq DNA Polymerase (Promega), 0.5 μl primer forward, 0.5 μl primer reverse and 3 μl Milli-Q water. To separate the bands of the different alleles, 10 μl of PCR product was run on a 2% agarose electrophoresis gel.

### Locomotion and survival assays

Locomotion experiments were performed on zebrafish in accordance with what was described in the study by Sztal *et al.*^24^ For locomotion assays at 6 dpf, the distance travelled above the inactivity threshold of 1 mm/s and below the maximum burst threshold of 30 mm/s was quantified in a 10-min period using the ZebraLab software (Viewpoint Life Sciences). The primary researcher was blinded to genotype during data acquisition and tracking analysis. After the completion of the experiment, fish were anesthetized using 0.0016% tricaine methanesulfonate (Sigma) in embryo media, and DNA was extracted and utilized for genotyping. Genotyping was performed after tracking analysis for a statistical comparison of maximum acceleration at 2 dpf and the distance travelled at 6 dpf between *uba5* mutants and wild-type siblings.

For long-term analyses of locomotor function and survival, 5-day-old zebrafish were anesthetized using 0.0016% tricaine methanesulfonate (Sigma) in embryo media, DNA was extracted from a small tail clipping that was used to genotype embryos. At 7 dpf, fish were transferred to individual tanks according to their genotype. A maximum of 25 fish were raised per genotype, with the number of surviving fish recorded daily. Fish were monitored daily to ensure that fish suffering from disease were humanely killed (>300 mg/l tricane methanesulfonate) before disease resulted in emaciation due to failure to feed. Locomotion analyses were performed weekly for a 10-min period as described above. At 2 and 3 weeks post fertilization, fish were transferred to 24-well and 6-well plates, respectively, and the distance travelled was tracked in Zebraboxes (Viewpoint). From 4 weeks post fertilization, fish were transferred to individual tanks, placed in Zebracubes (Viewpoint) and allowed to acclimatize for 5 min in darkness before tracking. The primary researcher was blinded to genotype during data acquisition, and each fish was randomly allocated to a position on the 24- or 6-well plates, Zebraboxes and Zebracubes using a list randomizer online tool (https://www.random.org).

### cDNA synthesis and reverse transcription-PCR

For reverse transcription-PCR (RT-PCR), total RNA was extracted from whole embryos using TRIzol reagent (Invitrogen Life Technologies). cDNA was synthesized from 1 µg of RNA in a 20 μl reaction using a Protoscript first-strand cDNA synthesis kit (New England Biosciences) and oligo(dT)20 and Random Primer Mix primers following the supplier’s instructions. The primers used for RT-PCR were *uba5*F: GCAGGACTTCTTCCCAAGCA, *uba5*R: GGCTGGGTG TAGACAAGCAT, *uba5*utrF: ACACCAACCCGAGTTTAGCA, uba5ex1F: GAGCGCCGAAGTCGTAGATT, *uba5*ex3R: CCACAGCAAACGAGCGGATT, *uba5*ex4R: TTGGCCAGCTCCACCTTATC, *uba5*ex9F: TGCAGGACTTCTTCCCAAGC, *uba5*ex11R: CCTGATGCGTCCTGTAGCTC, *β-actinF:* GCATTGTGACCGTATGCAG and *β-actinR:* GATCCACATCTGCTGGAAGGT GG.

### Whole-mount *in situ* hybridization and immunohistochemistry

To generate the *uba5* RNA probe, an *uba5* fragment was amplified from cDNA using the RT-PCR primers described above and cloned into pGEM-T easy (Promega). The probe template was amplified from the plasmid using *uba5* reverse (GGCTGGGTG TAGACAAGCAT) and M13 forward (TGTAAAACGACGGCCAGT) primers, and the antisense RNA probe was then generated using digoxygenin and T7 RNA polymerase (Invitrogen). Whole-mount *in situ* hybridization was performed on wild-type embryos in accordance with what was described in the study by Ruparelia *et al.*^25^ Images were acquired using an Olympus SZX10 dissecting microscope at 50× and 80× magnification, and image stacks were processed using the Zerene Stacker software (https://zerenesystems.com) to create an extended depth of focus.

Immunohistochemistry was carried out at 6 dpf in accordance with what was described in the study by Sztal *et al.*^21^ The antibodies used were anti-acetylated α-tubulin (Sigma, T6793, 1/1000), Alexa Fluor-488 (Molecular Probes, 1/150) and Alexa Fluor-594 phalloidin (ThermoFisher Scientific, 1/150). Embryos were embedded in 1% low melting point agarose (A9414; Sigma) on 0.8 mm fluorinated ethylene propylene (FEP) tubing (Bola) and images were acquired using a Thorlabs confocal microscope equipped with an Olympus 20× water dipping 1.0 NA objective. Maximum intensity projections were generated using Fiji (http://fiji.sc).

### Western blotting

Protein samples were extracted in RIPA buffer and quantified using the Qubit3 Fluorometer (Invitrogen). Thirty micrograms of each sample, along with 1× reducing agent (ThermoFisher Scientific) and 1× protein loading dye (ThermoFisher Scientific), was heated at 95°C for 10 min and the proteins were separated on a NuPAGE 4–12% Bis-Tris gel. The antibodies used were anti-Ufm1 (Abcam, ab108062, 1/1000; Abcam, ab109305, 1/1000), anti-UBA5 (Abcam, ab177507, 1/1000), anti-NCAM (Merck Millipore, AB5032, 1:1000), anti-Pink1 (Cell Signaling Technology, 6946, 1:1000), anti-mouse and anti-rabbit HRP-conjugated secondary antibodies (Southern Biotech, 1/10 000). Immunoblots were developed using ECL prime (GE healthcare) and total protein staining was performed with Direct Blue solution (Sigma). Protein levels in each sample were normalized against total protein using ImageLab software (Bio-Rad), and values for each genotype were normalized to wild-type values to allow a comparison between biological replicates.

### Electron microscopy

Zebrafish were fixed in 2.5% glutaraldehyde/2% PFA in a 0.1 M sodium cacodylate buffer (pH 7.4) overnight and post-fixed in an osmium buffer [1% OsO4/1.5% K3Fe (III)(CN), pH 7.4] for 2 h at 4°C in the dark. Dehydration was performed with ethanol and embedding was done in Epon 812 epoxy resin. After polymerization, ultrathin sections of 70 nm were cut using a diamond knife (Ultra 45° Diatome) on a Leica Ultracut UCT7, placed on 50 mesh copper grids with a carbon-coated formvar support film and stained with uranyl acetate and Waltons lead citrate. High-resolution EM imaging was done on a Jeol1400Flash TEM at 80 keV. All electron microscopy imaging procedures were completed at the Ramaciotti Centre for Cryo-Electron Microscopy (Monash University, Melbourne, Australia).

### Body length analysis

For body length analysis at 5 dpf, embryos were anesthetized using 0.0016% tricaine methanesulfonate (Sigma) in embryo media and lateral images were acquired using a SZX16 microscope with an Olympus 4× 1.0 NA objective. For body length analysis at later developmental stages, images were captured from the Zebrabox and Zebracube videos using Adobe Premiere Pro version 15.2.0. Body length was measured by drawing a polyline from the most anterior point of the body to the most posterior point of the tail, using Fiji (http://fiji.sc).

Following image acquisition, DNA was extracted from 5 dpf embryos and used for genotyping. Animals at later development stages were previously genotyped for locomotion and survival assays. In both experiments, the primary researcher was blinded to genotype during body length measurement, with the genotypes revealed by a second researcher for a statistical comparison of the body length between *uba5* mutants and wild-type siblings.

### Brain imaging and analysis

*uba5^+/−^* fish were crossed to the Tg(*Huc:EGFP*) strain^26^ and raised to adulthood. Tg*(Huc:EGFP); uba5^+/−^* fish were crossed to *uba5^+/−^* fish, and their progeny was raised in embryo media containing *N*-phenylthiourea 200 µM (PTU, Sigma) from 12 h to suppress pigment formation. Embryos were sorted for GFP fluorescence at 1 dpf. At 4 dpf, the embryos were anesthetized using 0.0016% tricaine methanesulfonate (Sigma) in embryo media and DNA was extracted from a small tail clipping and was used to genotype embryos. At 6 dpf, the embryos were anesthetized and set in 1% low melting point agarose in E3 containing 0.0016% tricaine methanesulfonate (Sigma) in 0.8 mm FEP tubing (Bola). Images were taken using a Thorlabs confocal microscope with an Olympus 20× water dipping 1.0 NA objective, pinhole 25 µm, 2 µm/pixel, step size = 1 µm, averaging = 16 frames. Brain image registration analysis was performed using the Advance Normalization Tools registration software (3.0.0.0) in accordance with what was described in the studies by Dark *et al.*^27^ and by Gupta et al.^28^ Total brain and total cerebellar volume, which includes the eminentia granularis, valvula cerebelli, corpus cerebelli and cerebellar neuropil regions, were examined. During image acquisition and analysis, primary researchers were blinded to genotype, with the genotypes later revealed by another researcher for a statistical comparison between *uba5* mutants and wild-type siblings.

### Statistics

Statistical analyses were performed with GraphPad Prism version 8.3.1 (332) and SPSS version 26 (IBM). Changes in Ufm1 and Ncam1 protein levels between the different genotypes were normalized to wild-type values, and a one-way ANOVA, followed by pairwise comparison using Dunnett’s test, was used to test for a reduction in protein levels in mutant fish compared to wild-type siblings. For swimming data analyses, outliers (>3× the interquartile range) were removed, and statistical significance was assessed with a linear model using likelihood estimation with replicate and genotype as fixed effects. For body length analyses, outliers were removed using the ROUT method, and a one-way ANOVA, followed by pairwise comparison using Dunnett’s test, was used to test for a reduction in the body length of the mutant fish compared with wild type. For brain volume analyses, outliers (>3× the interquartile range) were removed and statistical significance was assessed with a mixed model using genotype as a fixed effect and replicate as a random effect. Pairwise comparisons of groups were performed using the least significant difference. To compare the survival distribution of wild-type and *uba5* mutant fish, a log-rank (Mantel–Cox) test was used.

Most experiments were performed in embryonic and larval stages prior to 7 dpf, when the sex of the animals is not possible to determine. Additionally, in the survival assay, since *uba5* mutants died before sex could be determined, the effect of sex on the variable analysed was not assessed.

## Results

### *Uba5* is expressed during zebrafish development

Zebrafish have a single *UBA5* orthologue with conserved synteny (Supplementary Fig. 1A), encoding a protein with 80% identity and 90% similarity to the human protein. This indicates that UBA5 structural and functional properties are likely conserved in zebrafish. To determine the temporal expression of zebrafish *uba5*, we performed RT-PCR analysis on total RNA isolated from whole wild-type embryos ranging from 1 to 6 days post fertilization (dpf). We detected that *uba5* is expressed at all developmental stages examined (Supplementary Fig. 1B). To determine the spatial expression pattern of *uba5*, we next performed whole-mount *in situ* hybridization and found that *uba5* is expressed in the midbrain-hindbrain boundary, hindbrain and somite boundaries at 1 dpf, and it is detected throughout the brain and in the developing spinal cord at 2 dpf (Supplementary Fig. 1C). This pattern of expression is consistent with a role for Uba5 in the developing nervous system.

### *Uba5* mutant strains have a loss of wild-type *Uba5*

**Figure 1:**
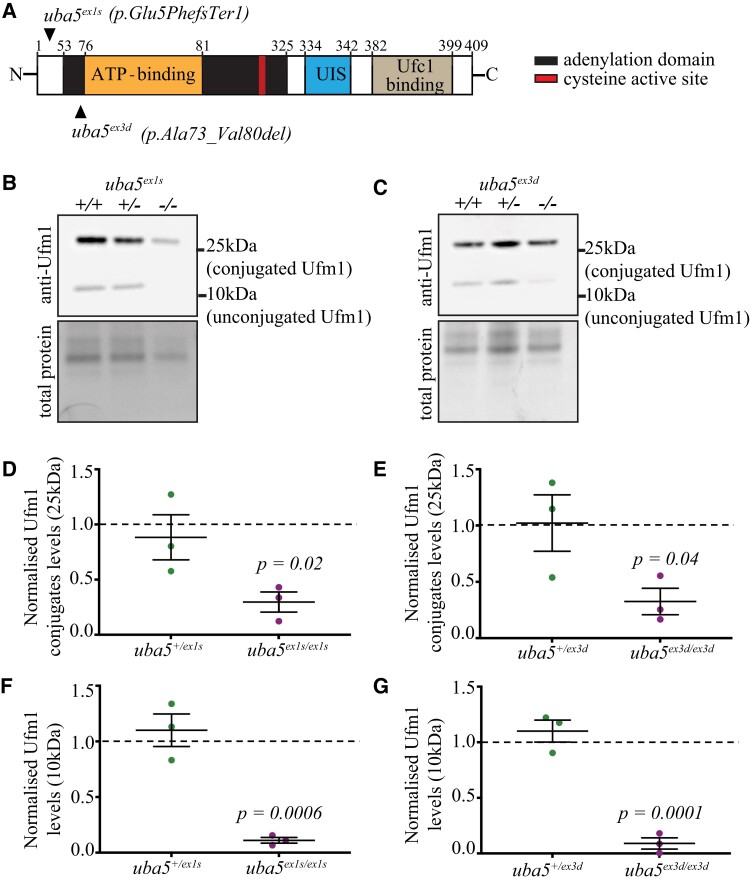
**Ufm1 and Ufm1-conjugate levels are reduced in *uba5^−/−^* fish at 6 dpf.** (**A**) Schematic of Uba5 wild-type protein and mutation sites in zebrafish. The mutant strains were generated using CRISPR/Cas9 genome editing. The *uba5*^ex1s^ strain carries a 73-base pair insertion following the ATG, resulting in a frameshift and incorporation of a premature stop after 5 amino acids, whereas the *uba5*^ex3d^ strain carries a 24-base pair deletion that removes the ATP-binding domain. (**B–G**) Western blot for Ufm1 and Direct Blue staining for total protein (loading control) on protein lysates obtained at 6 dpf. Ufm1 and Ufm1-conjugate levels were normalized to that of the respective total protein levels. The changes in Ufm1 and Ufm1-conjugate levels in *uba5* mutants were determined relative to the levels of wild-type siblings. Statistical significance was calculated using a one-way ANOVA with Dunnett’s *post hoc* multiple comparison correction test. Data are presented as mean ± SEM for three independent biological replicates, each consisting of a pooled sample of 20–25 embryos. UIS, Ufm1-interacting sequence; Ufc1, ubiquitin-fold modifier conjugating enzyme 1; Ufm1, ubiquitin-fold modifier 1.

To examine the consequence of Uba5 loss, we generated two independent *uba5* mutant zebrafish strains, one with a nonsense mutation in exon 1 [*c.10_11ins (73)*; *p.E5FfsTer1*; subsequently referred to as *uba5^ex1s^*] leading to a truncated and non-functional protein, and one with an in-frame deletion that removes the ATP binding domain in exon 3 (*c.214_237del*; *p.A73_V80del*; subsequently referred to as *uba5^ex3d^*), using CRISPR-Cas9 editing (Fig. 1A; Supplementary Fig. 2).

To determine whether the mutations result in *uba5* mRNA nonsense-mediated decay, we first performed RT-PCR analysis on total RNA isolated from 6 dpf embryos. We did not detect a reduction in transcripts in either *uba5*^ex1s/ex1s^ or *uba5*^ex3d/ex3d^ animals when compared to their wild-type siblings, indicating that *uba5* mutant mRNA is not degraded. A single band (∼183 bp) was amplified in *uba5*^ex3d/ex3d^ animals using primers spanning exon 3, indicating the deletion of the ATP binding domain. In *uba5*^ex1s/ex1s^ animals, using primers spanning exon 1, we observed the expected band of ∼334 bp and a very feint shorter, band (Supplementary Figs 3 and 4) that lacked 124 bp, including the first ATG in exon 1 and the 73-bp insertion, suggesting the use of a cryptic splice site. To determine whether the protein was lost in the mutant lines, we carried out a western blot analysis using a Uba5 polyclonal antibody. We identified a band of ∼50 kDa in wild-type and heterozygous fish, not detected in *uba5*^ex1s/ex1s^ or *uba5*^ex3d/ex3d^ animals, indicating that the Uba5 full-length isoform is absent. However, bands with higher and lower molecular weight were present in both *uba5* mutants (Supplementary Fig. 5), suggesting that isoforms from alternative start sites, or cryptic splicing, may form.

### Loss of Uba5 results in reduced *Ufm1*-conjugates

As previous studies using tissue samples from affected individuals observed reduced UFM1-conjugated proteins,^9^ we performed a western blot analysis to detect Ufm1. We found that Ufm1-conjugated proteins of ∼25 kDa were reduced in *uba5^−/−^* fish, indicating that ufmylation is impaired, at 6 dpf. Ufm1-conjugate levels were reduced to 30%, in *uba5* mutant animals and, interestingly, non-conjugated Ufm1 was also found to be reduced to 9–11% of that in *uba5^+/+^* siblings (Fig. 1B–G; Supplementary Fig. 6). Under non-reducing conditions, however, free Ufm1 was found increased in both *uba5* mutants, suggesting that Ufm1 conjugation is impaired (Supplementary Fig. 7). In addition, we detected a 3.7-fold increase (*P =* 0.021) in Ncam1 (Supplementary Figs 8 and 9), known to interact with UFC1 and endocytosed dependent on ufmylation,^16^ suggesting that there is a lower Ncam1 protein turnover rate in *uba5^−/−^* fish.

### *Uba5* mutants display impaired motor function and growth, and shorter lifespan

**Figure 2:**
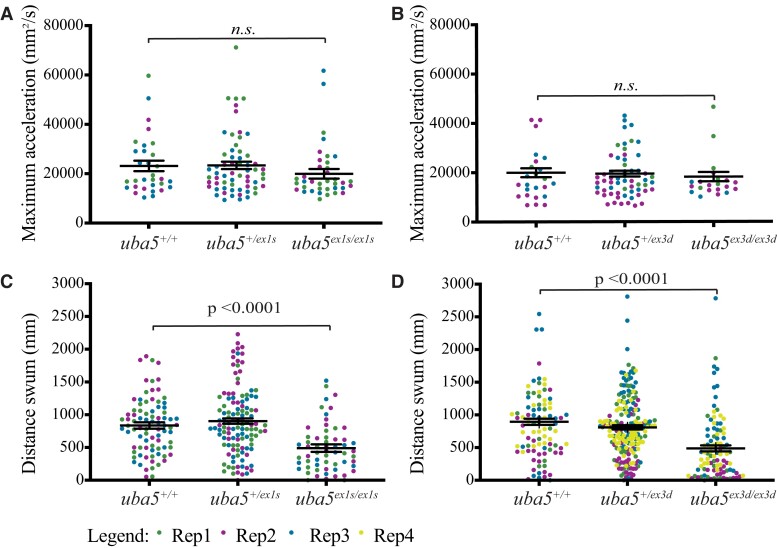
**Motor function in *uba5* mutants at 6 dpf. (A and B**) Quantification of the maximum acceleration recorded from touch-evoked response assays of *uba5*^ex1s/ex1s^ and *uba5*^ex3d/ex3d^ fish compared to wild-type siblings at 2 dpf revealed no significant differences. The error bars represent mean ± SD predicted by the linear model statistical test for three independent biological replicates (*uba5*^ex1s^ allele: *n* = 10, 7, 14 *uba5*^+/+^; *n* = 20, 18, 23 *uba5*^+/ex1s^; *n* = 17, 10, 10 *uba5*^ex1s/ex1s^. *uba5*^ex3d^ allele: *n* = 3, 12, 9 *uba5*^+/+^; *n* = 9, 25, 24 *uba5*^+/ex3d^; *n* = 6, 11, 4 *uba5*^ex3d/ex3d^). (**C and D**) Quantification of the distance travelled by *uba5*^ex1s/ex1s^ and *uba5*^ex3d/ex3d^ revealed a significant reduction in motor activity at 6 dpf compared to wild-type siblings. The graphs depict raw data for three to four independent experiments plotted with the mean ± SD predicted by the linear model statistical test (*uba5*^ex1s^ allele: *n* = 31, 26, 26 *uba5*^+/+^; *n* = 38, 40, 40 *uba5*^+/ex1s^; *n* = 22, 21, 16 *uba5*^ex1s/ex1s^. *uba5*^ex3d^ allele: *n* = 17, 24, 24, 27 *uba5*^+/+^; *n* = 47, 38, 43, 71 *uba5*^+/ex3d^; *n* = 20, 22, 26, 29 *uba5*^ex3d/ex3d^). In each experiment, each dot represents an individual zebrafish. n.s., non-significant; Rep, replicate.

Most affected individuals exhibit impaired motor function; therefore, we first analysed the swimming performance of *uba5* mutants to determine whether motor function was affected. At 2 dpf, the maximum acceleration, which is proportional to muscle force, generated during a touch-evoked escape response in *uba5^−/−^* animals, was comparable with *uba5^+/+^* siblings (Fig. 2A and B). At 6 dpf, however, the *uba5* mutants showed a 41–45% reduction in the distance swum compared with the *uba5*^+/+^ siblings (Fig. 2C and D). To confirm whether the reduction in the distance swum was a consequence of the mutation and not due to random off-target mutations generated during CRISPR mutagenesis, we further examined the swimming performance of compound heterozygotes for both alleles (*uba5*^ex1s/ex3d^) and detected a significant reduction in the distance swum (Supplementary Fig. 10), which is in line with our previous observation.

**Figure 3:**
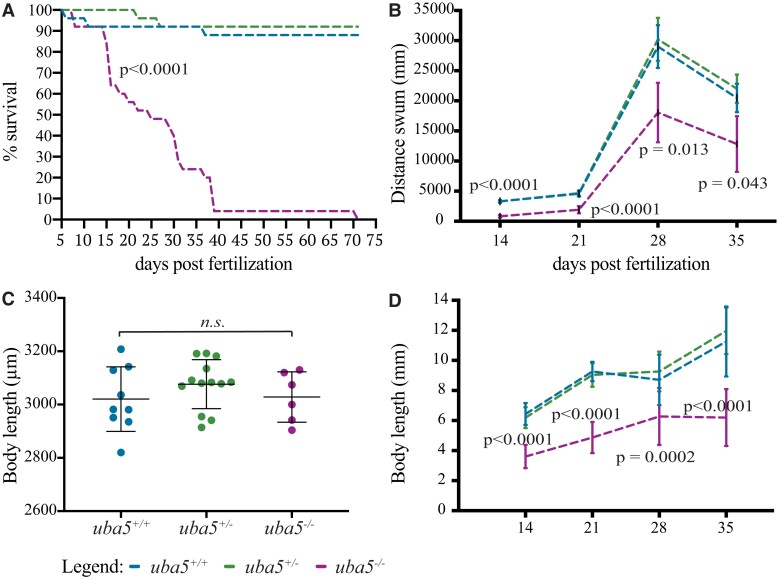
**Survival, motor function and growth are impaired in *uba5*^ex1s/ex1s^ fish.** (**A**) 25 *uba5*^+/+^, 25 *uba5*^+/ex1s^ and 25 *uba5*^ex1s/ex1s^ embryos were raised, and their survival was analysed. (**B**) Quantification of the distance travelled showed a significant reduction of the motor function in *uba5*^ex1s/ex1s^ compared to wild-type siblings. Data are represented as mean ± SD predicted by the linear model statistical test for one biological replicate analysed at 14 (*n* = 23 *uba5*^+/+^; *n* = 25 uba5^+/ex1s^; *n* = 23 *uba5*^ex1s/ex1s^), 21 (*n* = 23 *uba5*^+/+^; *n* = 25 *uba5*^+/ex1s^; *n* = 14 *uba5*^ex1s/ex1s^), 28 (*n* = 23 *uba5*^+/+^; *n* = 23 *uba5*^+/ex1s^; *n* = 12 *uba5*^ex1s/ex1s^) and 35 dpf (*n* = 23 *uba5*^+/+^; *n* = 23 *uba5*^+/ex1s^; *n* = 6 *uba5*^ex1s/ex1s^). (**C**) Measurement of the body length at 5 dpf did not reveal a significant difference between *uba5*^ex1s/ex1s^ and wild-type siblings (*n* = 9 *uba5*^+/+^; *n* = 13 *uba5*^ex1s/+^; *n* = 6 *uba5*^ex1s/ex1s^). Each dot represents an individual zebrafish. The error bars represent mean ± SD for one biological replicate. n.s., non-significant using a one-way ANOVA. (**D**) Measurement of the body length at 14 (*n* = 23 *uba5*^+/+^; *n* = 25 *uba5*^+/ex1s^; *n* = 23 *uba5*^ex1s/ex1s^), 21 (*n* = 21* *uba5*^+/+^; *n* = 25 *uba5*^+/ex1x^; *n* = 14 *uba5*^ex1s/ex1s^), 28 (*n* = 23 *uba5*^+/+^; *n* = 21* *uba5*^+/ex1s^; *n* = 12 *uba5*^ex1s/ex1s^) and 35 dpf (*n* = 23 *uba5*^+/+^; *n* = 23 *uba5*^+/ex1s^; *n* = 5* *uba5*^ex1s/ex1s^) demonstrates that *uba5^ex1s/ex1s^* are smaller than wild-type siblings at later stages of development. Data are represented as mean ± SD predicted by the linear model statistical test. *These numbers were originally the same as in (**B**), but we were unable to measure the body length of some fish, and therefore, these were not included in the analysis.

Because the majority of affected individuals fail to thrive and have a reduced lifespan, we monitored the motor function and survival of *uba5*^ex1s/ex1s^ mutants at later developmental stages. We found that the number of *uba5*^ex1s/ex1s^ mutant animals significantly decreased from 15 dpf, with 50% surviving up to 25 dpf and only 4% surviving from 39 dpf, and their maximum lifespan was 70 dpf (Fig. 3A). In comparison, wild-type fish exhibited 88% survival. An analysis of the swimming performance at later developmental stages demonstrated that, in line with the observations at 6 dpf, the *uba5*^ex1s/ex1s^ mutants show a significant reduction in the distance swum from 14 dpf, exhibiting <50% of the distance swum of *uba5*^+/+^ siblings throughout their lifespan (Fig. 3B).

Given that, in some cases, delayed growth has been reported, we also carried out an analysis of the body length of the *uba5*^ex1s/ex1s^ mutants to determine whether body growth was affected. At 5 dpf, the *uba5*^ex1s/ex1s^ mutants could not be phenotypically distinguished from the *uba5*^+/+^ siblings (*uba5*^+/+^ 3020 ± 121.1 µm, *uba5*^−/−^ 3028 ± 94.38 µm), excluding a growth deficit as the underlying cause of the motor function impairment observed at 6 dpf (Fig. 3C). However, the *uba5*^ex1s/ex1s^ animals were significantly smaller than the *uba5*^+/+^ siblings from 14 dpf (*uba5*^+/+^ 6.434 ± 0.729 mm, *uba5*^−/−^ 3.605 ± 0.774 mm), showing a 40–50% reduction in body length throughout their lifespan (Fig. 3D).

### Peripheral nerve damage is detected alongside motor dysfunction

**Figure 4:**
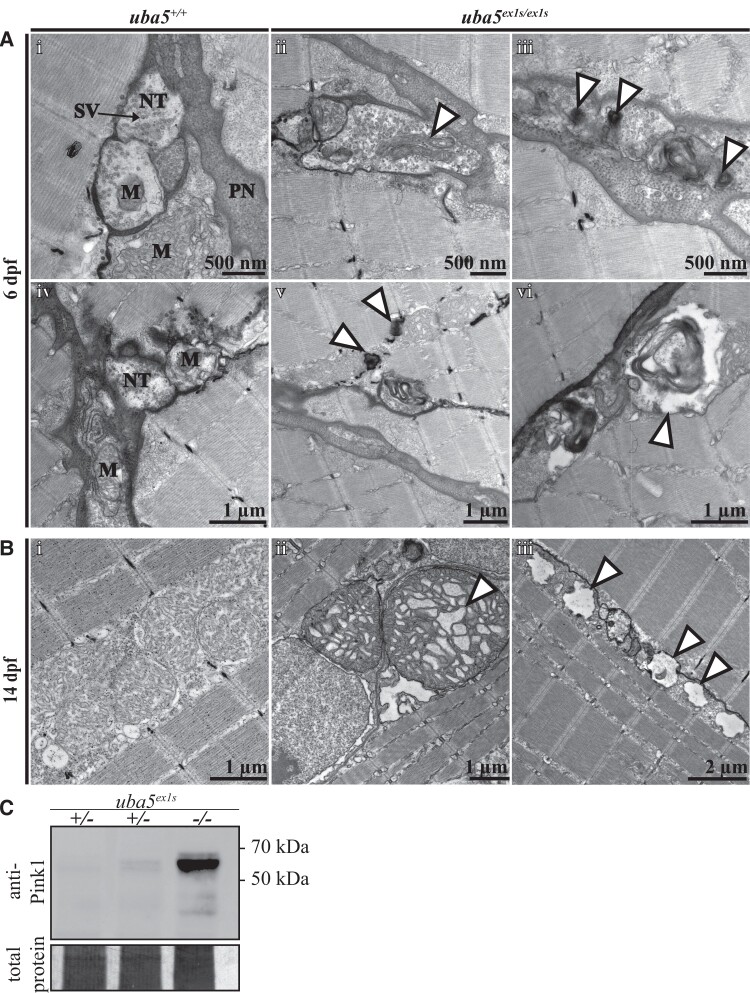
**Ultrastructural analysis of the peripheral nerves and skeletal muscle in *uba5*^ex1s/ex1s^.** (**A**) Patterning of the peripheral nerves and skeletal muscle at 6 dpf (i–vi). Electron microscopy images show mitochondrial abnormalities, including elongated mitochondria (ii) and degenerating mitochondria (iii), in the nerve terminals of *uba5*^ex1s/ex1s^ fish (arrowhead). Degradation vacuoles enclosing debris (vi), and autophagic structures associated with mitochondria in the skeletal muscle (v), are also observed. (**B**) Patterning of the skeletal muscle at 14 dpf shows abnormal mitochondria with swollen cristae (ii) and vacuolated mitochondria (iii) in *uba5* mutants (arrowhead). (**C**) Western blot for Pink1 and Direct Blue staining (loading control) on protein lysates obtained from pooled samples of 25 embryos at 6 dpf. M, mitochondria; NT, nerve terminal; PN, peripheral nerve; SV, synaptic vesicles.

To examine the neuromuscular changes caused by Uba5 deficiency, we used an anti-α-acetylated tubulin antibody to label mature neurons and their axonal tracts, and phalloidin to label actin in the muscle fibres, at 6 dpf. We did not detect noticeable differences in neuronal morphology or muscle integrity in *uba5*^ex1s/ex1s^ fish compared to *uba5*^+/+^ siblings at this time point (Supplementary Fig. 11). However, electron microscopic analyses revealed that *uba5*^ex1s/ex1s^ mutants exhibit abnormalities within the peripheral nerves at 6 dpf, with autophagic structures and large vesicles enclosing debris observed (Fig. 4A). Furthermore, we detected elongated mitochondria and ‘onion-ring’ double membranous structures at the peripheral nerve terminals, indicating mitochondrial degradation. In line with this finding, we also observed autophagic structures associated with mitochondria in the skeletal muscle, indicating that there is widespread mitochondrial damage in the *uba5*^ex1s/ex1s^ mutants (Fig. 4A). Mitochondrial damage was more striking within the skeletal muscle of the *uba5*^ex1s/ex1s^ mutants at 14 dpf, with enlarged mitochondria displaying abnormal cristae and adjacent mitochondria depleted of cristae undergoing vacuolar degeneration. Interestingly, the myofibril pattern in the *uba5*^ex1s/ex1s^ mutants was indistinguishable from wild-type siblings, indicating that the muscle structure was not affected (Fig. 4B).

Damaged mitochondria produce a signal that leads to an accumulation of the full-length isoform of PINK1 at the outer mitochondrial membrane, resulting in the recruitment of PARKIN and initiation of mitophagy.^29^ We used a polyclonal antibody to detect Pink1 levels in whole zebrafish lysates at 6 dpf. We found that there was an increase in full-length Pink1 isoform levels, observed as a high-molecular-weight band located between 50 and 70 kDa, in the *uba5*^ex1s/ex1s^ mutants (Fig. 4C; Supplementary Figs 12 and 13). This accumulation of Pink1 suggests that there is mitochondrial damage at this early stage of development.

### *Uba5* mutants display cerebellar neuronal damage

**Figure 5:**
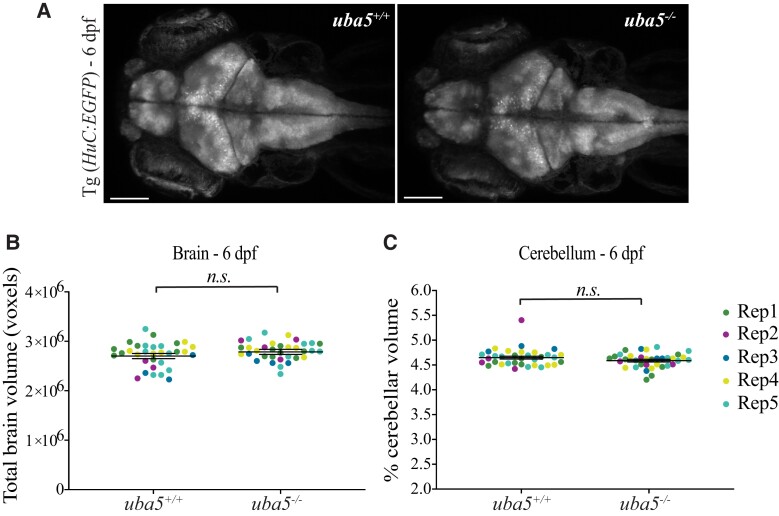
**Quantification of total brain and cerebellar volume in *uba5*^ex1s/ex1s^ animals at 6 dpf.** (**A**) Maximum intensity projections of whole brain from Tg (*Huc*:*EGFP*); *uba5*^+/+^ and Tg (*Huc*:*EGFP*); *uba5*^ex1s/ex1s^ 6 dpf zebrafish. (**B**) Total brain and (**C**) cerebellar volume analyses showed no significant changes in *uba5*^ex1s/ex1s^ (brain volume *n* = 9, 4, 4, 8, 9; cerebellar volume *n* = 10, 4, 4, 8, 9) compared to *uba5*^+/+^ (brain volume *n* = 9, 3, 3, 7, 10; cerebellar volume *n* = 8, 4, 3, 9, 10). Each dot represents an individual zebrafish. Data represents raw data for five biological replicates plotted with the mean ± SD predicted by the mixed model statistical test. Scale bar = 100 µm. n.s., non-significant; Rep, replicate.

**Figure 6:**
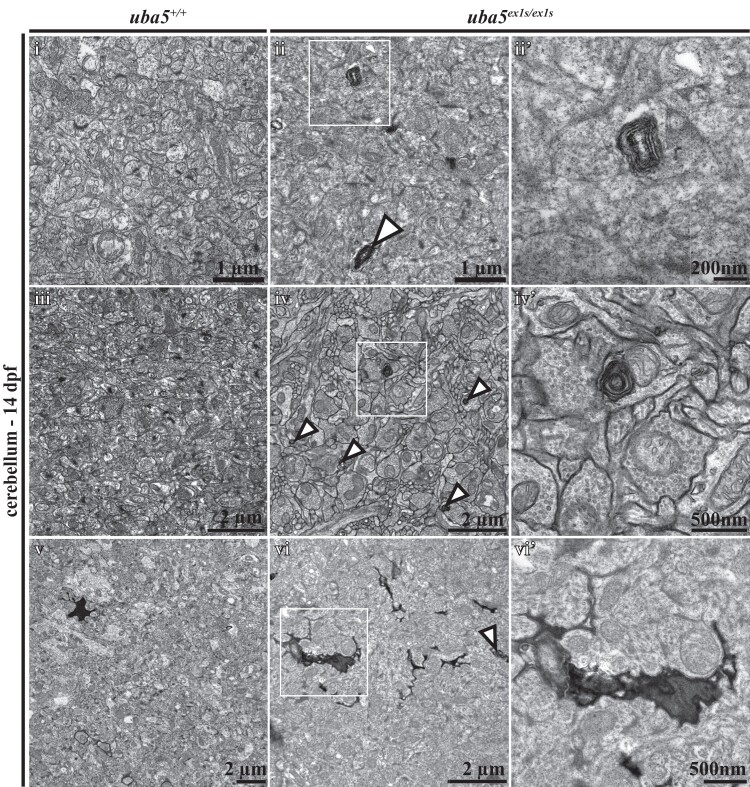
**Ultrastructural analysis of the cerebellum at 14 dpf shows abnormal findings in *uba5*^ex1s/ex1s^ animals.** Membranous swirls (ii, ii′), membrane whorls inside neurons that are indicative of mitochondrial degradation (iv, iv′) and cytoplasmatic condensation (vi, vi′) are observed in *uba5***^ex1s/ex1s^** mutants (arrowheads) compared to *uba5*^+/+^ (i, iii, v). (ii′, iv′, vi′). A higher magnification view of boxed areas.

Brain imaging has shown that, although the findings are variable, affected individuals often present with cerebral and/or cerebellar atrophy.^1-7,9,11^ We measured the volume of the brain and the cerebellum in *uba5*^ex1s/ex1s^ mutants at 6 dpf using a pan-neuronal fluorescent reporter strain [Tg(*Huc:EGFP*)] and live confocal microscopy. We did not detect significant changes in total brain volume (*uba5*^+/+^ 2 701 990 ± 52 230, *uba5*^−/−^ 2 786 120 ± 51 040 mean ± SEM, *P* = 0.1) or cerebellar volume (*uba5*^+/+^ 4.649 ± 0.028, *uba5*^−/−^ 4.589 ± 0.027 mean ± SEM, *P* = 0.131) in the *uba5*^ex1s/ex1s^ mutants compared to wild-type siblings at this time point (Fig. 5). An electron microscopic examination of the brain, however, revealed that *uba5*^ex1s/ex1s^ mutant fish exhibited abnormalities in the cerebellum at 14 dpf. Abnormal membranous swirls, appearing as small darkly stained ovals, were detected in the *uba5*^ex1s/ex1s^ mutants. Additionally, degenerating mitochondria, visible as ‘onion-ring’ structures with a double membrane, and dark cytoplasmic condensations were identified within cerebellar neurons, indicating neuronal death (Fig. 6).

## Discussion

Currently, there are no effective treatment options for individuals affected with *UBA5* disorders. This is in part due to a limited understanding of the role of UBA5 in neurons, the signal transduction pathways that are modulated by ufmylation and the complexity and variation of the disease pathophysiology. A vertebrate model that can recapitulate the core clinical symptoms of the disease and can be used to identify targetable pathways, and test potential therapeutic approaches, is therefore warranted. In this study, we generated and characterized novel *uba5* mutant zebrafish, demonstrating the suitability of these models to investigate the pathophysiology of *UBA5* disease.

To date, 24 variants in *UBA5* have been reported to cause neurological disease. Approximately 67% of these are located in the adenylation domain, 19% in the UFC1-binding domain and the C-terminus and 14% in the N-terminus of the UBA5 protein.^1-12^ Most missense variants result in mildly impaired UBA5 function; however, two N-terminus variants inherited in homozygosity were reported to cause severe forms of disease, leading to the death of the affected individuals within 16 days and 16 weeks of age, respectively.^8,12^

Here, we generated mutant strains with either an N-terminal nonsense mutation (*uba5*^ex1s^) after amino acid five or lacking the ATP-binding domain (*uba5*^ex3d^). While nonsense-mediated decay pathways were not activated and *uba5* transcripts still detected in both strains, western blot revealed that the full-length Uba5 isoform was absent from *uba5* mutants. However, bands with higher and lower molecular weights, not detected in wild-type or heterozygous siblings, were present in both *uba5*^ex1s/ex1s^ and *uba5*^ex3d/ex3d^ animals. Only one Uba5 isoform has been described in zebrafish; however, we suggest that alternative start sites could be utilised, which would lead to truncated forms of Uba5 in mutant animals. If this is the case, we would expect that the phenotypes would be severe, as in affected individuals carrying recessive N-terminal UBA5 variants.^8,12^ The loss of Uba5 function is further indicated by the increase in unconjugated Ufm1 and decrease in Ufm1 conjugates seen in both mutant strains.

We have shown that *uba5* mutant animals exhibit impaired motor function and reduced lifespan, consistent with phenotypes observed in affected individuals. In addition, while *uba5* mutants show a comparable body size to wild-type animals at early embryonic stages, they demonstrate generalized growth delay at later larval developmental stages. This is in line with the findings of previous mice studies that showed that *Uba5* mutants are smaller and appear developmentally delayed compared with their littermates during embryonic development.^19^ This is also in agreement with the growth impairment described in a number of affected individuals.^1,2,7,9,10^ At birth, body length and head circumference are within normal parameters in most affected individuals; however, in the first years of life, some individuals display progressive developmental delay with reduced head size and reduced body growth.^1,2,7,9,10^ In many affected individuals, disease progression is often accompanied by non-specific brain abnormalities, with cerebral and cerebellar atrophy being the most frequently reported.^1,2,4-7,9^ We recorded no changes in total brain and cerebellar volume in *uba5* mutants at 6 dpf. We did, however, detect signs of neuronal degeneration in the cerebellum of *uba5* mutants at 14 dpf, demonstrating that this brain region is affected at a later stage of development. In addition to brain and cerebellar atrophy, corpus callosum thinning is another recurrent neuroimaging finding observed in affected individuals.^1,3,5,6,9^ As zebrafish do not have a homologous structure to the human corpus callosum, however, we are not able to examine this brain structure.

Most affected individuals described so far exhibit clinical symptoms predominantly associated with the central nervous system.^1-11^ In 2020, Cabrera-Serrano *et al*.^12^ identified five individuals with severe sensory motor peripheral neuropathy associated with impaired motor function from the antenatal or neonatal period, demonstrating that *UBA5* deficiency can primarily affect the peripheral nervous system as well. More recently, Al-Saady *et al*.^1^ described one individual with a similar clinical phenotype to the epileptic encephalopathy affected individuals, exhibiting severe developmental delay, brain hypomyelination, reduced thalamic and cerebellar volume and refractory epilepsy, but also displaying motor peripheral neuropathy, demonstrating the involvement of both central and peripheral nervous systems in the disease pathophysiology. We have shown that the skeletal muscle and axonal tract morphology appear intact during early embryonic stages, but signs of active degradation, abnormal mitochondrial morphology and mitochondrial degeneration are detected in the peripheral nerves of *uba5* mutant fish at 6 dpf. Such observations revealed that peripheral nerve damage occurs at early stages and are in line with previous *uba5 Drosophila* knockdown studies that reported locomotor impairment associated with abnormal neuromuscular junctions at 3 days of age.^11^ While the contribution of peripheral nerve damage to the clinical presentation of the majority of affected individuals remains unknown, our observations therefore reinforce previous reports that defects in both peripheral and central nervous systems occur in response to UBA5 deficiency.

Notably, we have demonstrated that *uba5* mutant fish present mitochondrial abnormalities in the skeletal muscle without noticeable changes in muscle patterning, a phenotype that has not yet been reported in affected individuals or animal models. These, together with our observations of mitochondrial pathology in both central and peripheral nervous systems, and increased full-length Pink1 isoform levels at 6 dpf, revealed that Uba5 deficiency results in generalized mitochondrial damage, and that this damage is an early event associated with the disease phenotype. More importantly, these observations suggest that impaired skeletal muscle function due to abnormal mitochondria, which is independent of neuronal damage, may occur simultaneously.

Mitochondria have an indispensable role in ATP production, calcium signalling and metabolic pathways. Consequently, an accumulation of damaged mitochondria, due to an inability to eliminate excessive numbers or impaired mitophagy, often leads to neuronal damage and death.^30^ Although ufmylation has not yet been reported to have a direct role in mitochondria in vertebrates, it is important to note that studies in *Leishmania donovani* have demonstrated that Uba5, Ufc1 and Ufm1 are localized in mitochondria and that ufmylation is indispensable for the metabolism of mitochondrial fatty acids.^31,32^ As ufmylation is a highly conserved post-translational modification pathway, it might have a similar functional role in mitochondria of multicellular organisms, and therefore impaired ufmylation may result in dysfunctional mitochondria. Whether the mitochondrial damage observed in this study occurs as a direct consequence of impaired Uba5 function and ufmylation, or as a result of impaired downstream pathways, however, requires further investigation.

## Conclusion

Our study validates novel zebrafish models for *UBA5* deficiency that will help elucidate the molecular mechanisms underlying the pathophysiology of disease and can be utilized for high-throughput testing of potential therapeutic drugs. Importantly, we provide the first evidence that mitochondrial damage might contribute to the disease phenotype.

## Supplementary material

Supplementary material is available at *Brain Communications* online.

## Supplementary Material

fcad317_Supplementary_Data

## Data Availability

All raw data and statistics files are available on Bridges using the following link: https://bridges.monash.edu/projects/Genetic_model_of_UBA5_deficiency/147784.
