# Supplementary material for: Genetic model of UBA5 deficiency highlights the involvement of both peripheral and central nervous systems and identifies widespread mitochondrial abnormalities
Source: Brain Commun. 2023 Nov 20;5(6):fcad317. doi: 10.1093/braincomms/fcad317 (PMC10691876; doi:10.1093/braincomms/fcad317)
Supplement: fcad317_Supplementary_Data [file fcad317_supplementary_data.pdf]

Supplementary data for

Genetic model of UBA5 deficiency highlights involvement of both peripheral and central nervous systems and identifies widespread mitochondrial abnormalities

Rita J. Serrano[1], Viola Oorschot[2,4], Dashika Palipana[1], Vanessa Calcinotto[1], Carmen Sonntag[1], Georg Ramm[2,3], Robert J. Bryson-Richardson[1*]

[1] School of Biological Sciences, Monash University, Melbourne, Australia

[2] Monash Ramaciotti Centre for Cryo-Electron Microscopy, Monash University, Melbourne, Australia

[3] Department of Biochemistry and Molecular Biology, Biomedicine Discovery Institute, Monash University, Melbourne, Australia

[4] Current address: Electron Microscopy Core Facility, European Molecular Biology Laboratory, 69117, Heidelberg, Germany.

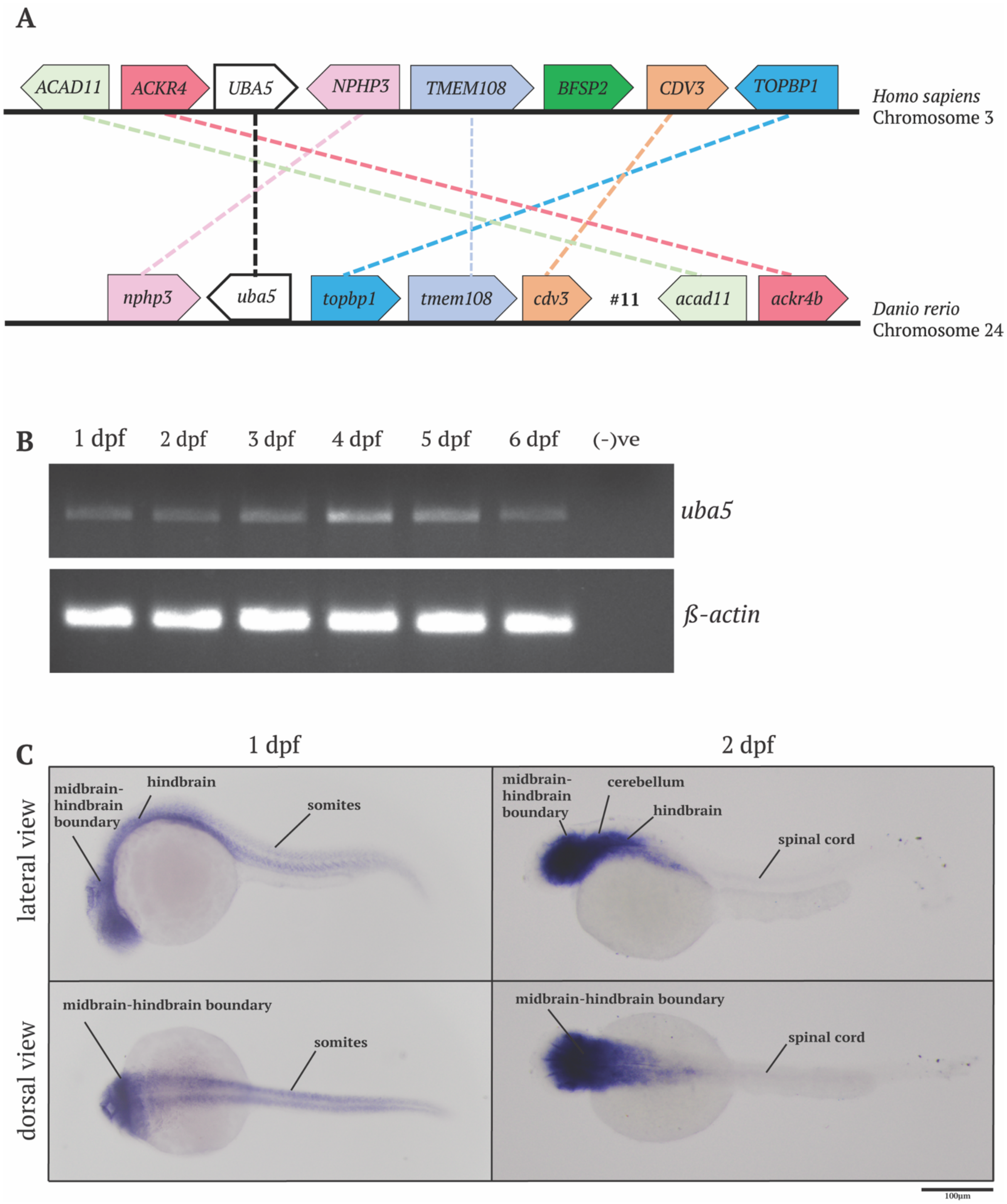

Supplementary Figure 1. Synteny and expression analysis of zebrafish *uba5*. (A) Genes flanking *UBA5* in the human genome and corresponding syntenic regions in zebrafish. The orientation of the arrowheads indicates the direction of transcription for each gene. Syntenic regions are shown in same color and with connecting dashed lines. # Indicates the number of genes not shown. (B) RT-PCR analysis of *uba5* expression in developing zebrafish reveals transcripts from 1 to 6 dpf. Expression of *β-actin* was used as a control. (C) *In situ* hybridization of *uba5* expression at 1 and 2 dpf.

allele *uba5*^{ex1s}

```
wt          ATGGCCACCG--------------------------------------------------
mutant      ATGGCCACCGTCTTTTAACTCTCGAATTTTACCACTCCCGAGCTTACAGTATGGATTCGT
            **********

wt          -----------------------TTGAGGAACTCAAGCTGCGAATTCGTGAGTTAGAAAA
mutant      GAGTTAGAAAATCATTTCTCCCCTTGAGGAACTCAAGCTGCGAATTCGTGAGTTAGAAAA
                                   *************************************

wt          TGAACTAATCAAATCCAAACAGAAGCAAAGTGATGCTGAACACAATATCAGACCGAAAAT
mutant      TGAACTAATCAAATCCAAACAGAAGCAAAGTGATGCTGAACACAATATCAGACCGAAAAT
            ************************************************************

wt          TGAGCAAATGAGCGCCGAAGTCGTAGATTCAAATCCATATAG
mutant      TGAGCAAATGAGCGCCGAAGTCGTAGATTCAAATCCATATAG (+73bp)
            ******************************************
```

allele *uba5*^{ex3d}

```
wt          AAAATCCGCTCGTTTGCTGTGGCGGTGGTGGGAGTCGGTGGGGTGGGAGTGTCACTGCA
mutant      AAAATCCGCTCGTTTGCT------------------------GTGGGAGTGTCACTGCA
            ******************                        *****************

wt          GAAATGCTCACCAGATGTGGCATTGGTAAG
mutant      GAAATGCTCACCAGATGTGGCATTGGTAAG (-24bp)
            ******************************
```

Supplementary Figure 2. Alignment of wild type and mutant *uba5* DNA sequences. The mutant sequences were obtained through sanger sequencing and aligned with the *uba5* wild-type sequence using ClustalW. There is a 73-bp insertion in the *uba5*^{ex1s} allele and a deletion of 24-bp in the *uba5*^{ex3d} allele compared to *uba5* wildtype. The asterisks denote the conserved residues and dashed lines the absence of residues in *uba5* wildtype and mutant sequences.

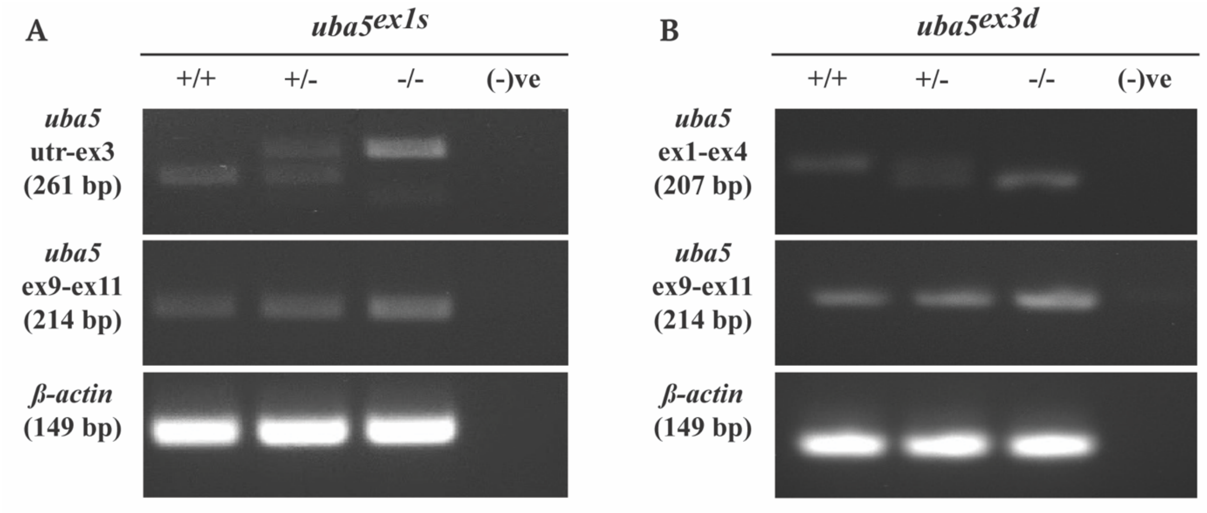

Supplementary Figure 3. *uba5* mRNA expression analyses on 6 dpf embryos from both *uba5* strains. (A) In *uba5*$^{ex1s/ex1s}$ animals, RT-PCR using primers spanning exon 1 amplified a longer transcript than in wild-type siblings due to the 73-bp insertion and a very feint shorter band. RT-PCR using primers spanning exon 10 showed that *uba5* transcripts are not absent from *uba5*$^{ex1s/ex1s}$ animals. (B) In *uba5*$^{ex3d/ex3d}$ animals, RT-PCR using primers spanning exon 3 detected a shorter transcript due to the 24-bp deletion. Similarly, RT-PCR using primers spanning exon 10 demonstrated that *uba5* transcripts are not absent from *uba5*$^{ex3d/ex3d}$ animals. *ß-actin* was amplified as a positive control.

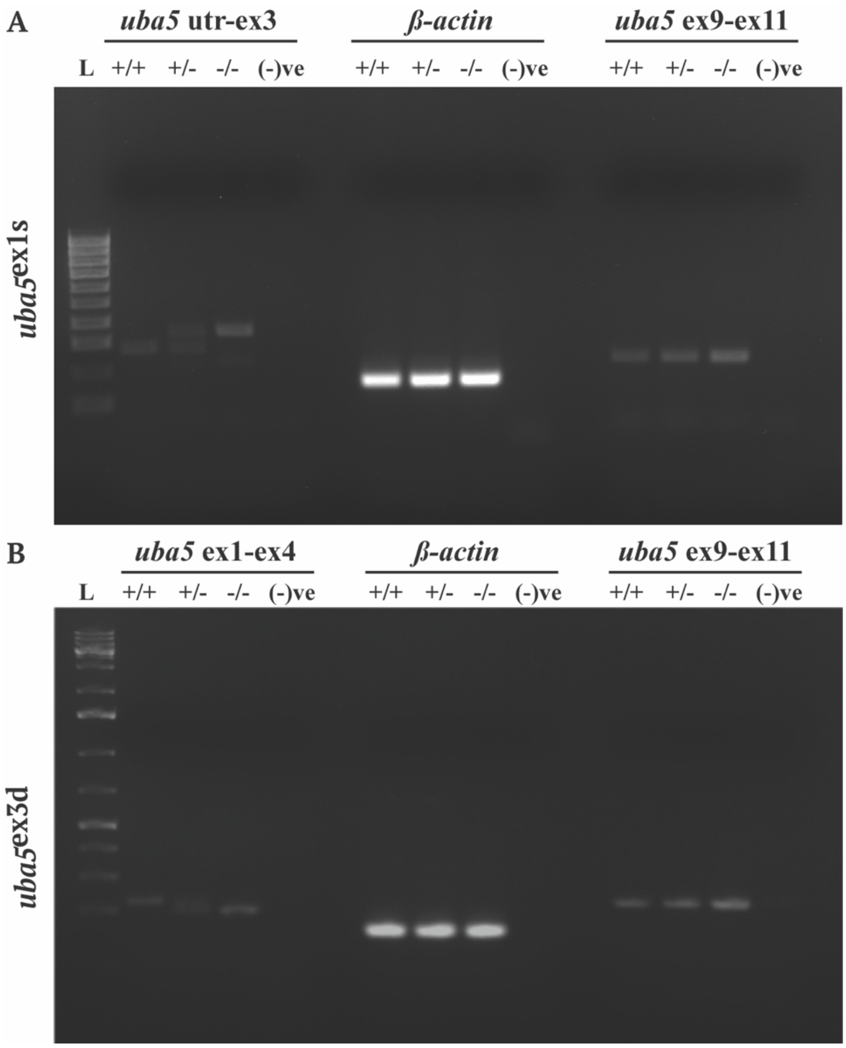

Supplementary Figure 4. RT-PCR performed on 6 dpf embryos from both *uba5* strains. Uncropped 2% agarose gel images (depicted in Figure S3) showing presence of *uba5* and *β-actin* transcripts in wild-type, heterozygotes and *uba5* mutant embryos. *β-actin* was used as a positive control.

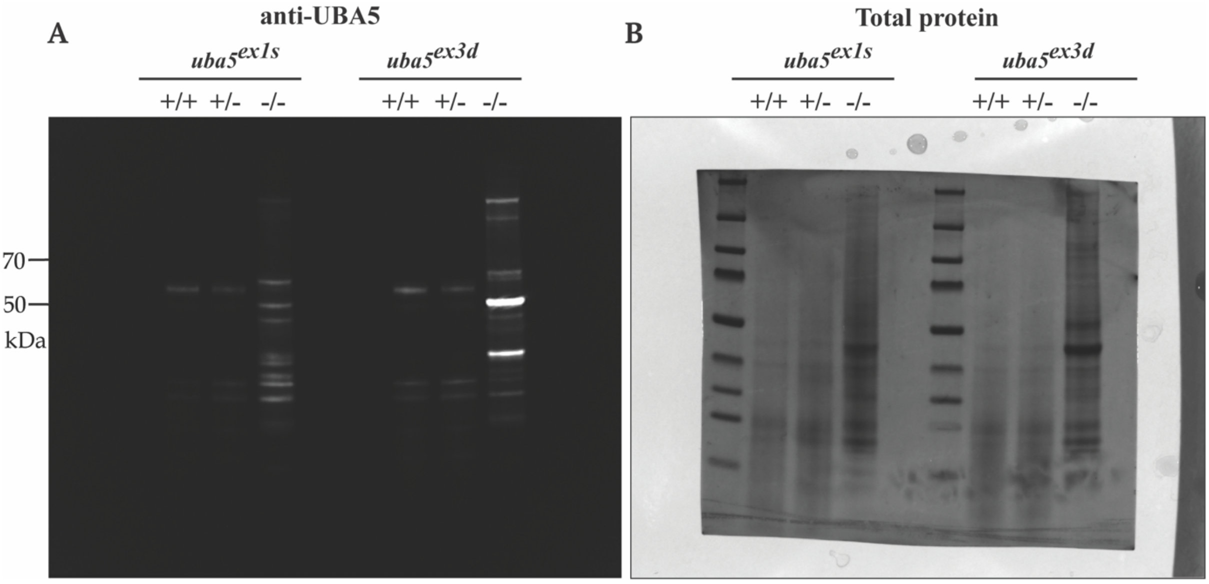

Supplementary Figure 5. Uba5 western blot analysis on 6 dpf embryos. (A) Western blot analysis showed that wild-type and heterozygous animals present a band at approximately 50 kDa that is absent in *uba5* mutants. However, bands of higher and lower molecular weight are detected in *uba5*$^{ex1s/ex1s}$ and *uba5*$^{ex3d/ex3d}$, suggesting alternative Uba5 isoforms are present. (B) Total protein analysis using direct blue staining was carried out as positive control. Spectra Multicolor Broad Range Protein Ladder (ThermoFisher Scientific, 26634) was used.

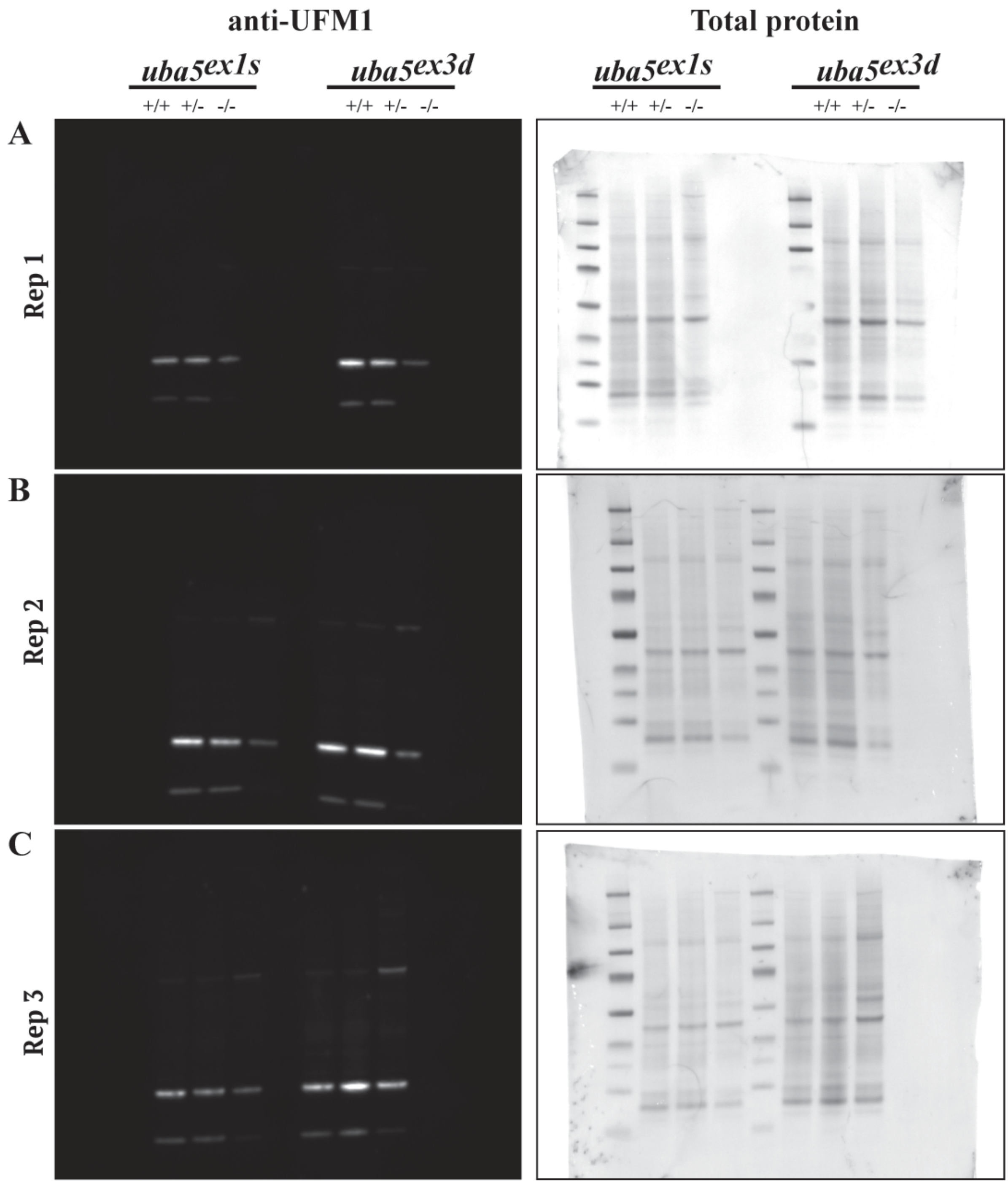

Supplementary Figure 6. Ufm1 western blot analysis (reducing conditions) on 6 dpf embryos. (A-C) Uncropped western blots showing reduced free Ufm1 (~10 kDa) and ~25 kDa Ufm1 conjugated proteins in *uba5* mutants compared to wild-type siblings. *uba5*^{ex1s} Rep2 and *uba5*^{ex3d} Rep3 blots are depicted in Figure 1B and 1C. Total protein analysis with direct blue staining was performed as positive control. Spectra Multicolor Broad Range Protein Ladder (ThermoFisher Scientific, 26634) was used.

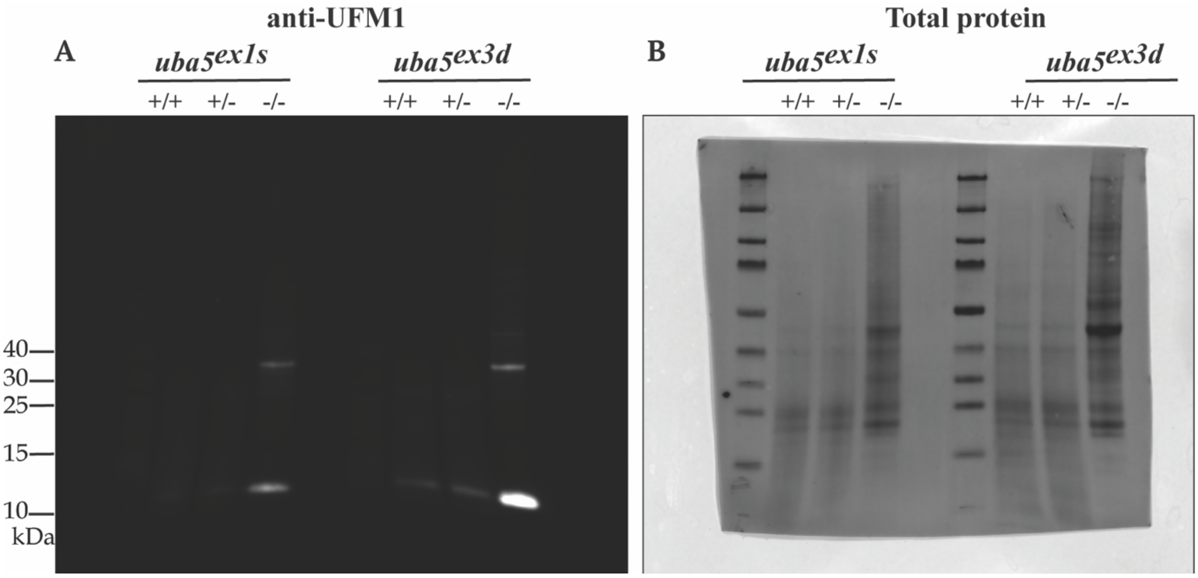

Supplementary Figure 7. Ufm1 western blot analysis performed under non-reducing conditions on 6 dpf embryos. (A) Western blot analysis showed increased unconjugated Ufm1 (~10 kDa) in *uba5* mutants compared to wild-type siblings. (B) Total protein analysis using direct blue staining was performed as positive control. Spectra Multicolor Broad Range Protein Ladder (ThermoFisher Scientific, 26634) was used.

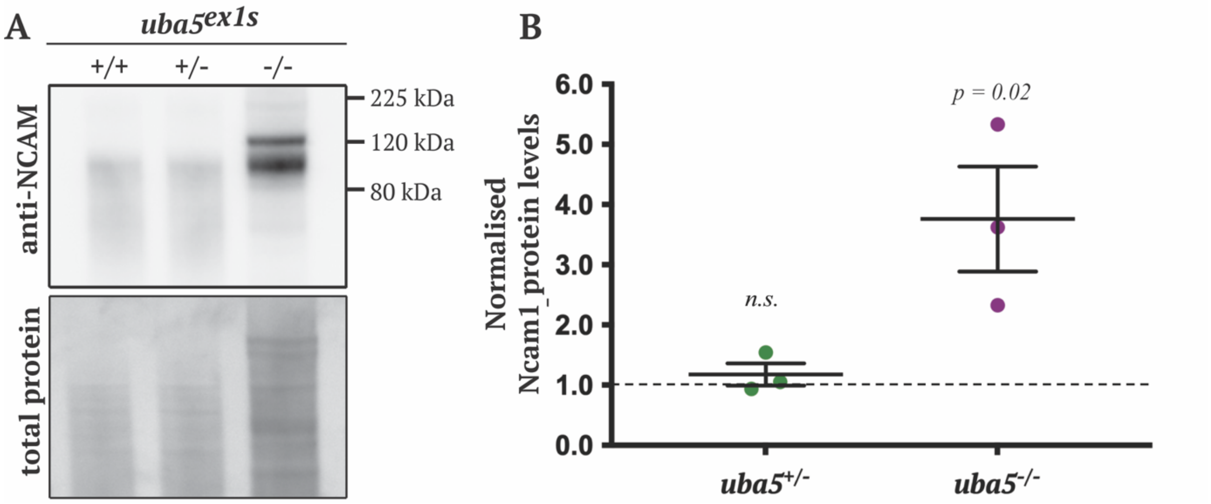

Supplementary Figure 8. Western blot analysis of Neural cell adhesion molecule 1 (Ncam1) in 6 dpf whole embryo tissue homogenates. (A) Representative western blot for Ncam1. (B) Comparison of Ncam1 levels in *uba5*$^{ex1s/ex1s}$ and wild-type siblings. All values were normalized to total protein, and then normalized to wild-type values, indicated by the dotted line. Statistical significance was calculated using a one-way ANOVA with Dunnett's post-hoc multiple comparison correction test. Data are represented as mean ± SEM for 3 biological replicates each consisting of a pooled sample of 20-25 embryos. *n.s.* non-significant.

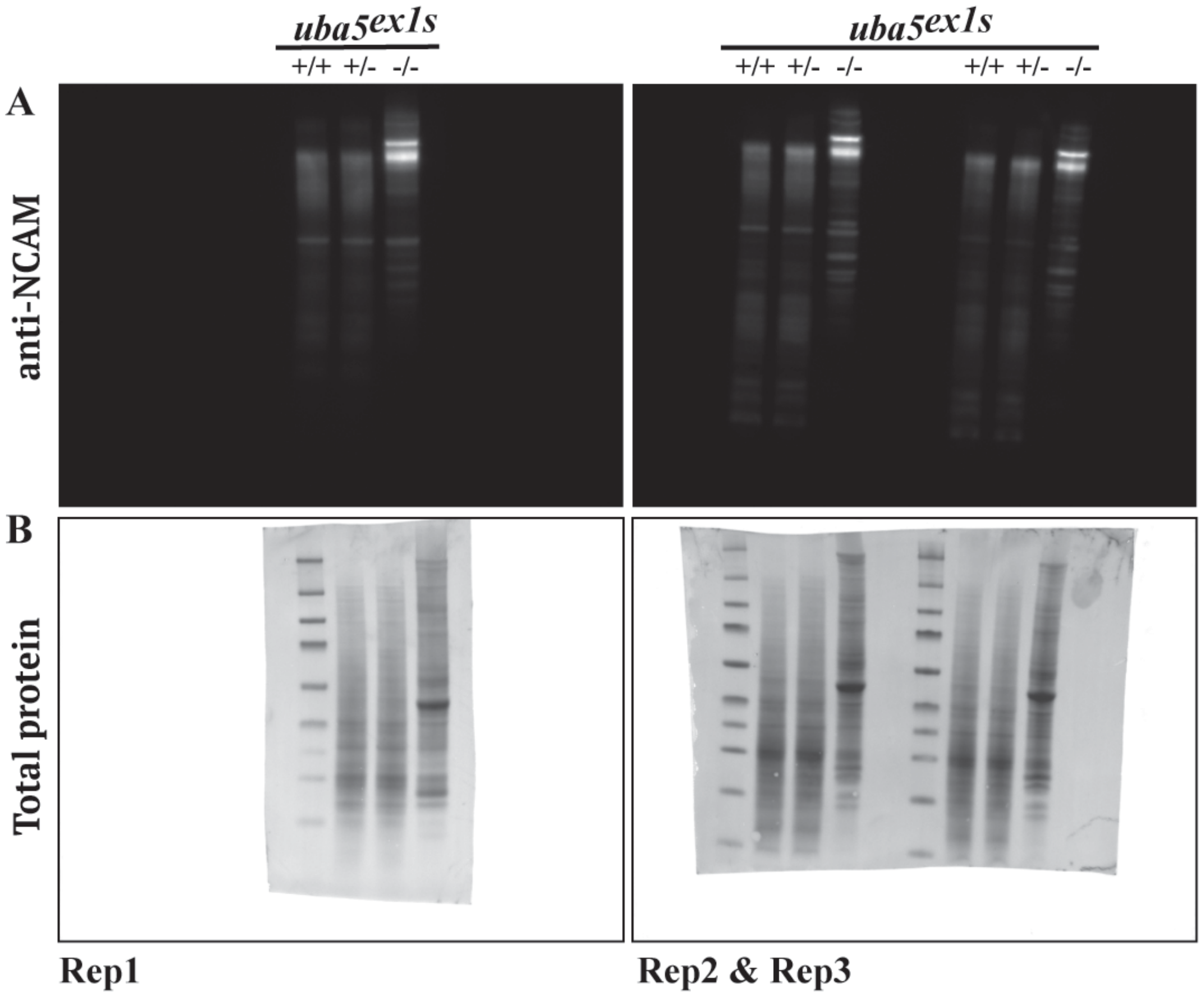

Supplementary Figure 9. Ncam1 western blots performed on 6 dpf whole embryo tissue homogenates. (A) Uncropped western blots showing increased levels of Ncam1 (bands within 80 and 120 kDa) in *uba5*$^{ex1s/ex1s}$ compared to wild-type siblings. Rep1 blot is depicted in Figure S8. (B) Total protein analysis was performed with direct blue staining. Spectra Multicolor Broad Range Protein Ladder (ThermoFisher Scientific, 26634) was used.

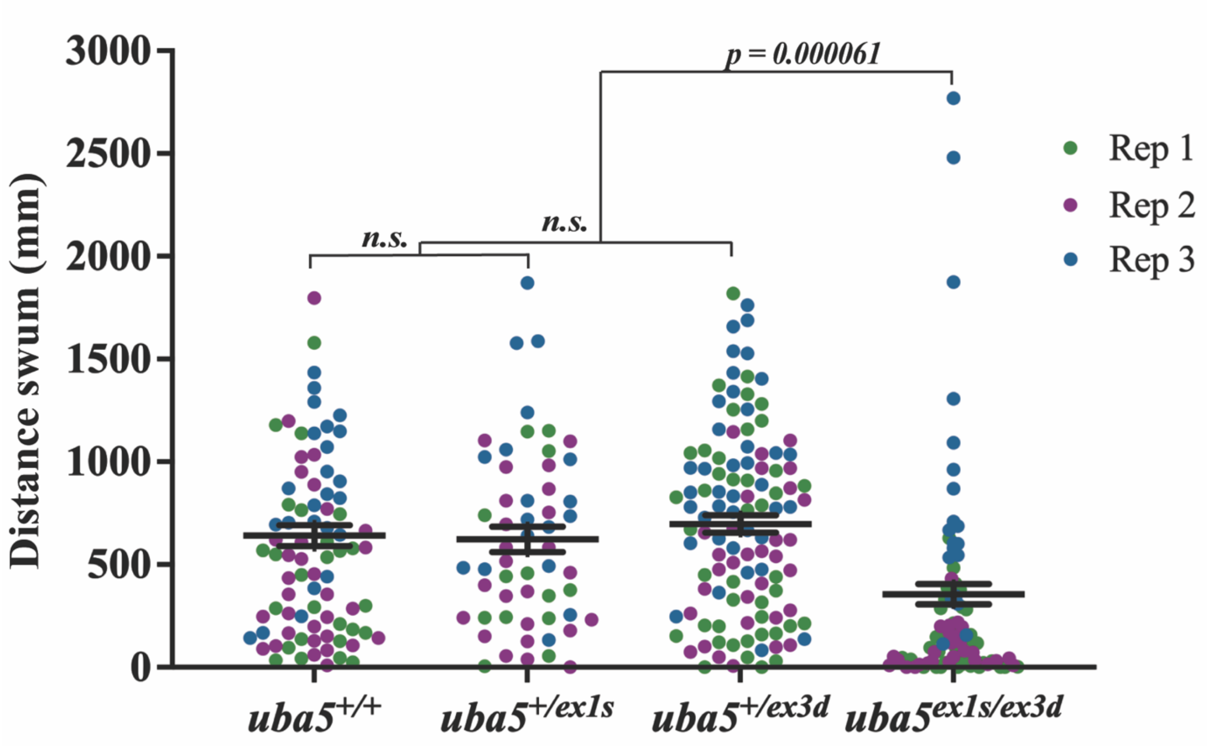

Supplementary Figure 10. Compound heterozygous *uba5*$^{\mathrm{ex1s/ex3d}}$ exhibit reduced locomotor function at 6 dpf. Quantification of the distance swum in *uba5*$^{\mathrm{ex1s/ex3d}}$ in comparison with wild-type siblings. The graph represents raw data for three independent experiments plotted with the mean ± SD predicted by the linear model statistical analysis (n = 26, 32, 25 *uba5*$^{+/+}$; n = 13, 24, 18 *uba5*$^{\mathrm{ex1s/+;}}$ n = 41, 32, 39 *uba5*$^{\mathrm{ex3d/+}}$; n = 37, 29, 21 *uba5*$^{\mathrm{ex1s/ex3d}}$). Each dot represents an individual zebrafish. *n.s.* non-significant.

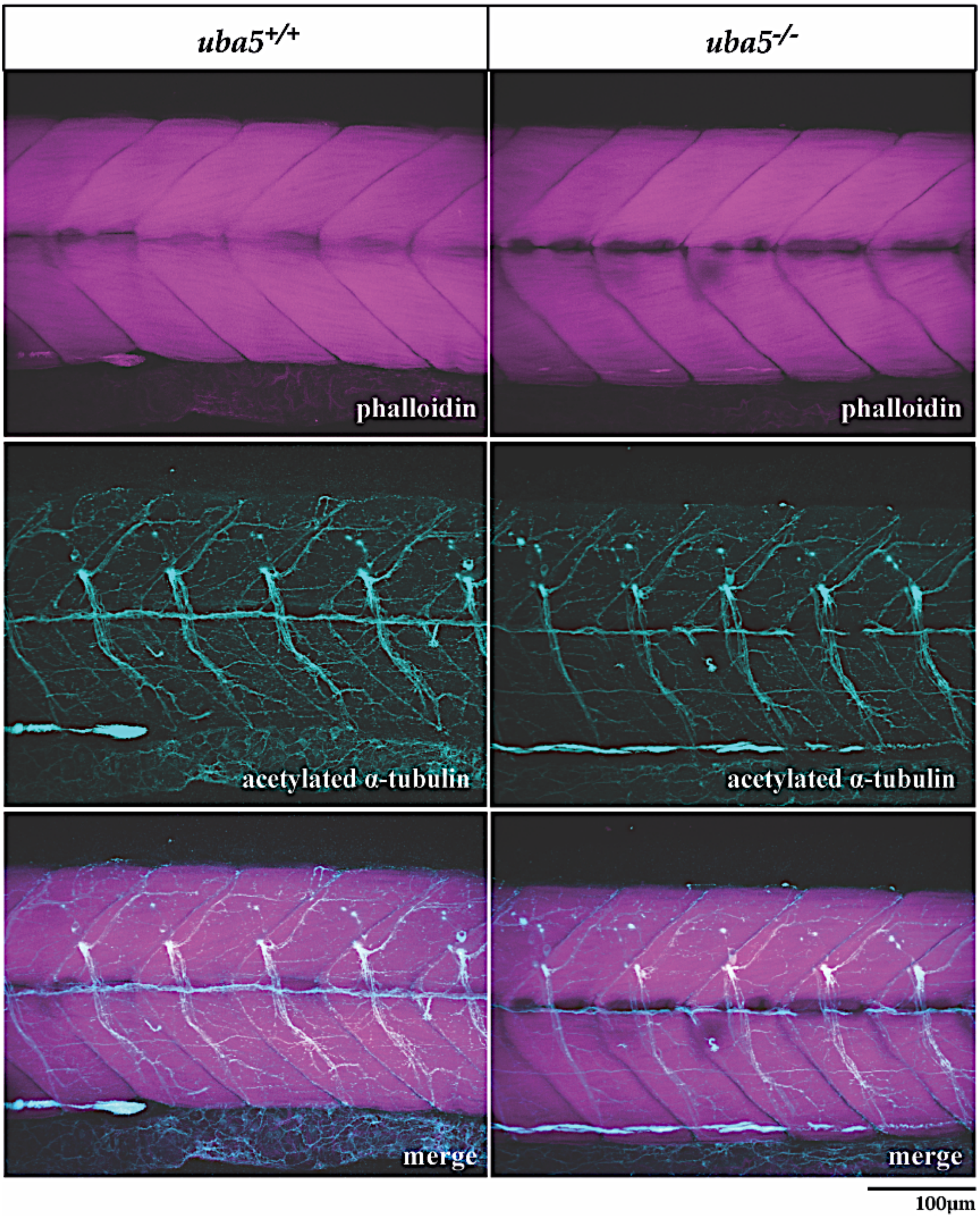

Supplementary Figure 11. The axonal network and muscle morphology are not overtly affected in *uba5*$^{ex1s/ex1s}$ at 6 dpf. Maximum projections of confocal z-series to visualize the muscle and axonal morphology in *uba5*$^{ex1s/ex1s}$ and wild-type siblings. Anti-α-acetylated tubulin (cyan) labels the axonal tracts of mature neurons and phalloidin (magenta) labels actin in the muscle fibers.

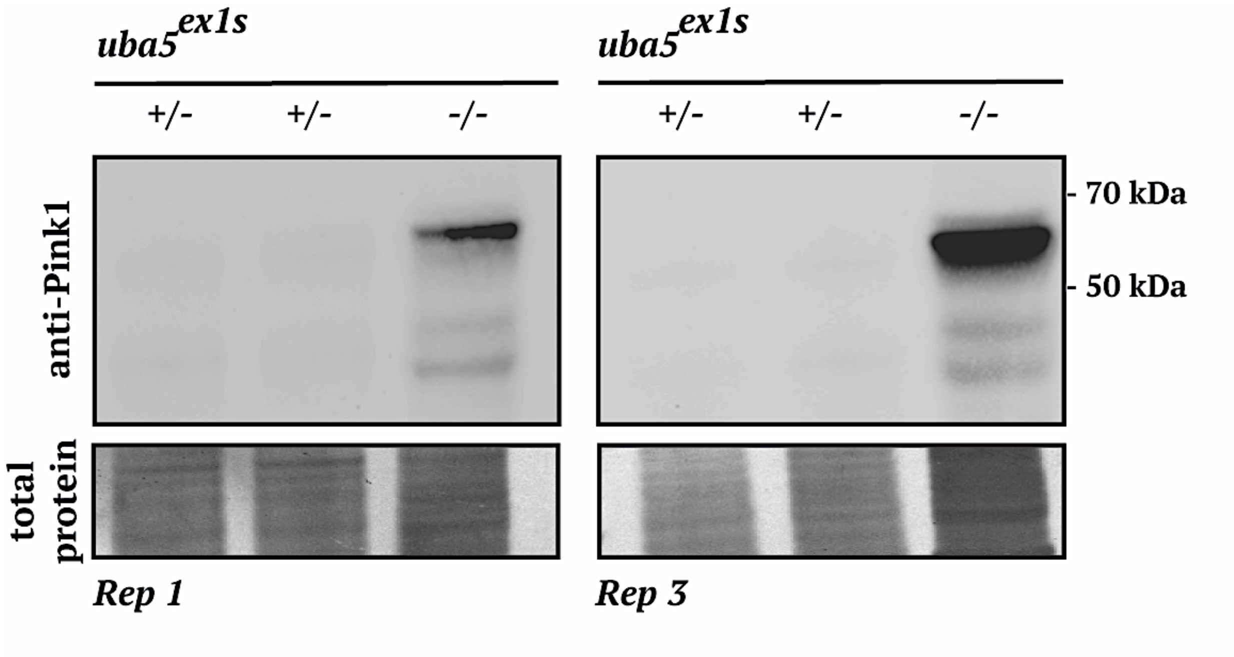

Supplementary Figure 12. Western blot analysis in whole embryo tissue homogenates shows accumulation of Pink1 in *uba5*$^{ex1s/ex1s}$ compared to wild-type siblings at 6 dpf. Each replicate consists of a pooled sample of 20-25 embryos.

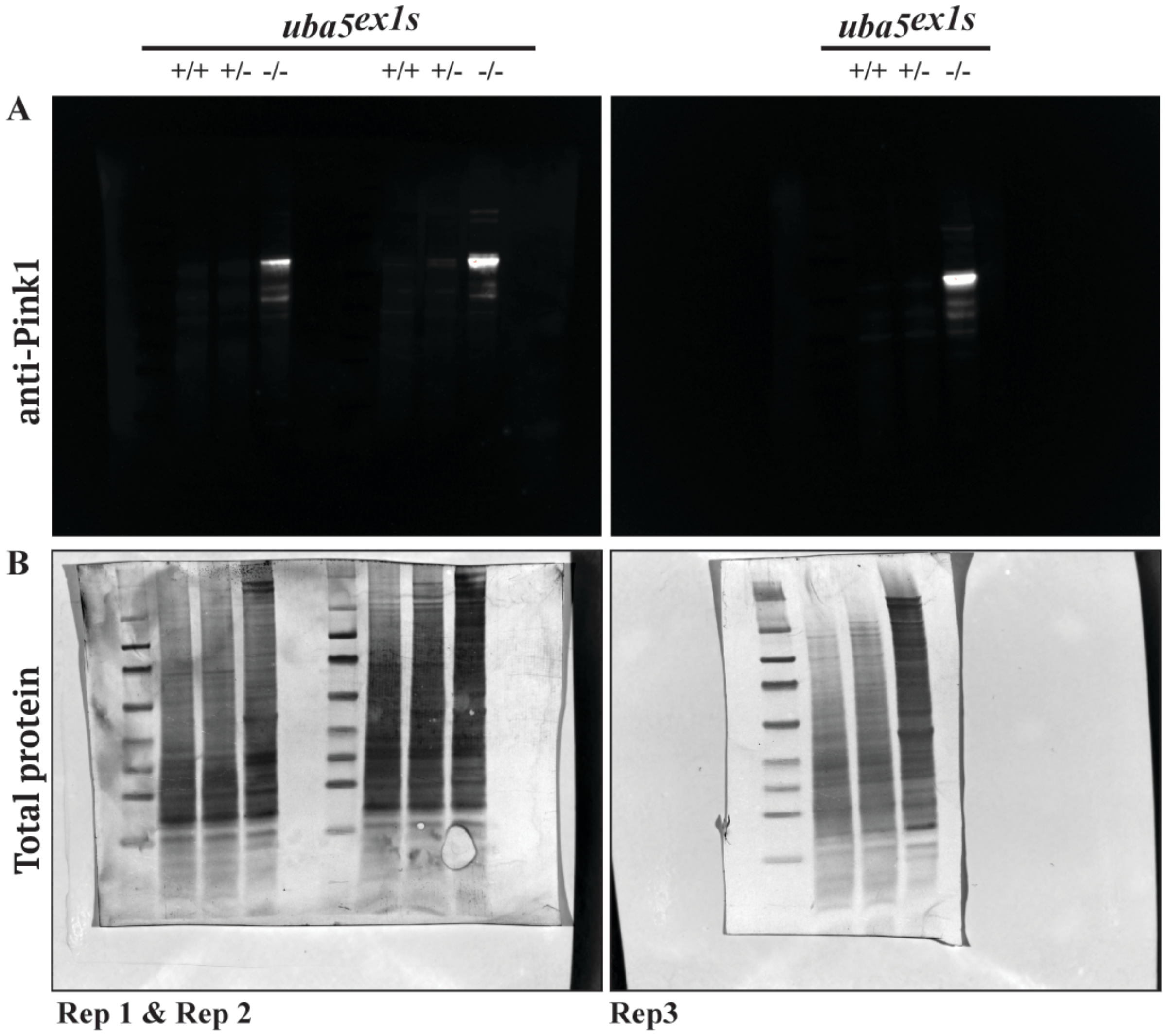

Supplementary Figure 13. Pink1 western blots performed on 6 dpf whole embryo tissue homogenates. (A) Uncropped western blots showing increased levels of Pink1 (bands within 50 and 70 kDa) in *uba5*$^{ex1s/ex1s}$ compared to wild-type siblings. Rep2 blot is depicted in Figure 4C. (B)Total protein analysis using direct blue staining was performed as positive control. Spectra Multicolor Broad Range Protein Ladder (ThermoFisher Scientific, 26634) was used.
